# NHE1 Protein in Repetitive Mild TBI-Mediated Neuroinflammation and Neurological Function Impairment

**DOI:** 10.3390/antiox13070836

**Published:** 2024-07-13

**Authors:** John P. Bielanin, Shamseldin A. H. Metwally, Helena C. M. Oft, Satya S. Paruchuri, Lin Lin, Okan Capuk, Nicholas D. Pennock, Shanshan Song, Dandan Sun

**Affiliations:** 1 Department of Neurology, University of Pittsburgh, Pittsburgh, PA 15213, USA; bielanin@pitt.edu (J.P.B.); smetwally@pitt.edu (S.A.H.M.); oft.helena@medstudent.pitt.edu (H.C.M.O.); paruchuriss@vcu.edu (S.S.P.); linlin5@pitt.edu (L.L.); capuk@pitt.edu (O.C.); ndp35@pitt.edu (N.D.P.); songs2@upmc.edu (S.S.); 2 Pittsburgh Institute for Neurodegenerative Disorders, University of Pittsburgh, Pittsburgh, PA 15213, USA; 3 Veterans Affairs Pittsburgh Health Care System, Pittsburgh, PA 15213, USA

**Keywords:** concussion, diffuse axonal injury, neuroinflammation, oxidative stress, white matter injury

## Abstract

Mild traumatic brain injuries (mTBIs) are highly prevalent and can lead to chronic behavioral and cognitive deficits often associated with the development of neurodegenerative diseases. Oxidative stress and formation of reactive oxygen species (ROS) have been implicated in mTBI-mediated axonal injury and pathogenesis. However, the underlying mechanisms and contributing factors are not completely understood. In this study, we explore these pathogenic mechanisms utilizing a murine model of repetitive mTBI (r-mTBI) involving five closed-skull concussions in young adult C57BL/6J mice. We observed a significant elevation of Na^+^/H^+^ exchanger protein (NHE1) expression in GFAP^+^ reactive astrocytes, IBA1^+^ microglia, and OLIG2^+^ oligodendrocytes across various brain regions (including the cerebral cortex, corpus callosum, and hippocampus) after r-mTBI. This elevation was accompanied by astrogliosis, microgliosis, and the accumulation of amyloid precursor protein (APP). Mice subjected to r-mTBI displayed impaired motor learning and spatial memory. However, post-r-mTBI administration of a potent NHE1 inhibitor, HOE642, attenuated locomotor and cognitive functional deficits as well as pathological signatures of gliosis, oxidative stress, axonal damage, and white matter damage. These findings indicate NHE1 upregulation plays a role in r-mTBI-induced oxidative stress, axonal damage, and gliosis, suggesting NHE1 may be a promising therapeutic target to alleviate mTBI-induced injuries and restore neurological function.

## 1. Introduction

Epidemiological research indicates that 70–90% of all traumatic brain injuries (TBIs) are mild and that these injuries are common among professional athletes engaged in contact and collision sports and military personnel [1]. Mild TBIs (mTBIs) are characterized by a transient disturbance in brain function with short-lived neurological symptoms, such as headache, dizziness, and confusion, in the context of normal neuroimaging results (i.e., CT scan) [2,3,4]. However, 10–25% of mTBI patients develop persistent post-concussion symptoms, which are associated with long-term cognitive deficits and white matter damage [3,5,6]. The underlying mechanisms of mTBI are not well defined, and currently, no effective treatment is available for mTBI-related pathogenesis.

Diffuse axonal injury (DAI), a key pathology after mTBI, is characterized by axonal stretching, mitochondrial swelling, cytoskeletal disorganization, and transport dysfunction that are accompanied by the accumulation of amyloid precursor protein (APP) [7,8]. Moreover, astrocyte reactivity and microglial activation are critical early responses to TBI-induced extracellular changes [4,9]. These cells exert complex, heterogeneous responses, including altered gene expression, hypertrophy, proliferation, and secretion of cytokines to regulate inflammation and limit tissue damage [10]. Oxidative stress and the formation of reactive oxygen species (ROS), mediated by the pro-inflammatory microglia and astrocytes, exacerbate axonal injury, drive brain damage, and hinder brain repair and neurological functional recovery [4,7,8].

Prior research has unveiled that stimulation of the Na^+^/H^+^ exchanger isoform 1 (NHE1), a vital pH-regulatory plasma membrane protein which facilitates the efflux of H^+^ in exchange for the influx of Na^+^, is instrumental in maintaining optimal intracellular pH (pH_i_) homeostasis [9]. This process is also essential for continuous activation of NADPH oxidase (NOX2) and the release of cytokines in neurons [9,11], proinflammatory microglia [9], and reactive astrocytes [12]. In our recent study, we reported that selective deletion of the microglial NHE1 protein in *Cx3cr1-Cre^ER+/−^;Nhe1^flox/flox^* mice reduced neuroinflammation, enhanced remyelination, and improved neurological functional outcomes in a moderate-TBI mouse model with open-skull injury [9]. However, whether pathological stimulation of NHE1 protein expression and activity plays a role in oxidative stress and the development of pathogenesis associated with repetitive mTBI (r-mTBI) remains unexplored.

In this current study, we investigated r-mTBI-induced changes in the expression of the NHE1 protein, axonal damage markers, neuroinflammation, and oxidative stress. Additionally, we tested the efficacy of post-r-mTBI administration of the NHE1 selective inhibitor, HOE642, in reducing brain injury and sensorimotor and cognitive deficits. Our findings reveal that post-r-mTBI pharmacological inhibition of the NHE1 protein in C57BL/6J mice led to a reduction in gliosis, axonal damage, oxidative stress, and MRI diffusion tensor imaging (DTI)-detected white matter damage. Moreover, this intervention resulted in improved locomotor and cognitive functional recovery. Thus, targeting the NHE1 protein may serve as a potential therapeutic strategy with antioxidant benefits for reducing mTBI pathology and improving neurological function.

## 2. Materials and Methods

### 2.1. Animals

All animal experiments described in this study were approved by the University of Pittsburgh Institutional Animal Care and Use Committee and adhered to the National Institutes of Health Guide for the Care and Use of Laboratory Animals. Additionally, all studies were reported in accordance with the Animal Research: Reporting In Vivo Experiments (ARRIVE) guidelines [13]. Food and water were provided to animals ad libitum and animals were housed in a temperature-controlled environment with 12/12 h light-dark cycles. All efforts were made to minimize the number of animals used for experiments and any animal suffering. The number of animals used per figure can be found in Table S1. Adult C57BL/6J wild-type (WT) mice (male, 2–3 months old) were used in this study. For transgenic knockout study, *Cx3cr1-CreER^+/−^* control (Ctrl) mice and *Cx3cr1-CreER^+/−^; Nhe1^f/f^* conditional knockout (*Nhe1* cKO) mice were used as described in our previous study (male and female, 2–3 months old) [9]. See Supplemental Materials for detailed methods.

### 2.2. Repetitive Mild (r-mTBI) Procedures

Adult C57BL/6J WT or transgenic mice were anesthetized using 1.5% isoflurane as previously described [9]. Mice were placed on a stereotaxic frame mounted with a controlled cortical impact (CCI) device (Leica Biosystems, Deer Park, IL, USA). Mice were impacted at 5 m/s with a 5 mm blunt tip, with a strike depth of 1 mm and a dwell time of 200 ms, mimicking an mTBI [14]. Repetitive injuries occurred on days 0, 2, 4, 6, and 8 for a total of 5 impacts with an inter-mTBI interval of 48 h. Stepwise changes in righting time and apnea time (indicators of neurological functional recovery) after each impact were recorded to detect animal-to-animal differences in response to CCI [14]. Apnea time refers to the number of seconds before a mouse resumes normal breathing after an impact. Righting time refers to the duration of time until the mouse returns to the upright position after the anesthesia was turned off. Sham animals underwent the same repeat procedures without receiving impacts. See Supplemental Materials for detailed methods.

### 2.3. Post-r-mTBI HOE642 Treatment

For the NHE1 inhibitor HOE642 (Cariporide, Sigma-Aldrich, St. Louis, MO, USA) study, C57BL/6J WT mice were used. HOE642 preparation and administration methods were the same as previously described [9]. HOE642 was dissolved at 1 mg/mL in dimethyl sulfoxide (DMSO) stock solution. Immediately before injection, the solution was diluted to 0.025 mg/mL in PBS. For the vehicle control (Veh), 2.5% DMSO in PBS was used. Veh or HOE642 (0.3 mg/kg body weight/day, i.p.) was administered twice daily for 7 days starting at 24 h after the 5th impact.

### 2.4. Behavioral Function Tests

Neurological functional impairments in mice were screened in a blinded manner with the rotarod accelerating test, open field test, y-maze spontaneous alternation test, and y-maze novel spatial recognition test. These tests were considered reliable for identifying and quantifying sensorimotor and cognitive impairments in rodent models [10,15,16,17], which were performed as previously described [9,10,16,17] with slight modification. Researchers were blinded to the surgical and/or treatment conditions of the individual animal throughout data collection and analyses. To account for baseline differences in motor learning performance on treatment outcome assessment, mice were trained on the rotarod apparatus for three days, running three trials per day, prior to surgery. Each mouse’s running time is recorded from trial start until 300 s elapses or they fall from the rotarod (whichever occurs first). All mice assigned to either Veh or HOE642 treatment group achieved an average running time of 300 s on the rod before they were subjected to rmTBI. Thus, potential influences of variability in the baseline characteristics and handling procedures on outcome analysis and conclusion were minimized. See Supplemental Materials for detailed methods.

### 2.5. MRI and DTI of Ex Vivo Brains

MRI and DTI procedures were performed as previously described [9,10]. At 60 days post-first-injury (dpi), the same cohort of mice that underwent behavioral assessments were humanely euthanized through CO_2_ overdose. Subsequently, they underwent transcardial perfusion with ice-cold 0.1M PBS (pH 7.4) followed by an infusion of 4% paraformaldehyde (PFA). The mice were then decapitated, keeping the brains intact within the skull to prevent anatomical distortion as previously outlined [9,10]. Regions of interest (ROIs) were delineated, segmenting the corpus callosum (CC), hippocampal CA1, internal capsule, and external capsule in both hemispheres from four scanned sections in each brain. Unlike standard MRI techniques such as T1 and T2, which view white matter as a uniform tissue, DTI offers precise details about the structural characteristics and directional alignment of white matter pathways [18]. Diffusion MRI enables the estimation of brain fiber structures by utilizing water diffusion properties as a probe. In an unorganized environment, water molecules diffuse freely in a random (Brownian) manner while an organized environment, like brain tissue, restricts water diffusion more readily along axons but prevents them from escaping or crossing axonal boundaries. This coherent directionality is termed anisotropic diffusion. By evaluating diffusivity across multiple directions, the diffusion tensor can be computed, facilitating the estimation of axon bundle orientations and integrity [19]. Fractional anisotropy (FA), mean diffusivity (MD), axial diffusivity (AD), and radial diffusivity (RD) values were then calculated for each ROI, employing previously described methodology [9,10]. See Supplemental Materials for detailed methods.

### 2.6. Immunofluorescence Staining and Analysis

Immunofluorescence staining procedures were similar to those used in previous studies [9,10,16,17]. Coronal sections (25 μm thickness, at the level 1.46 mm posterior to bregma) were used for immunostaining. Table S2 depicts the primary and secondary antibodies used in immunostaining. Negative controls were established by staining brain sections with secondary antibodies only (Figures S1 and S2). A minimum of three fluorescent images were captured for each region of interest using a 40× lens on a Nikon A1R inverted confocal laser-scanning microscope (Olympus, Tokyo, Japan). Identical digital imaging acquisition parameters were used for all images in a set, and images were obtained and analyzed in a blinded manner throughout this study. Field intensity was measured within the delineated regions and cell counts were measured by converting images into binary with semi-automated cell counting using consistent threshold parameters for specific cell types. See Supplemental Materials for detailed methods.

### 2.7. IMARIS 3D Reconstruction and Analysis

Z-stack analysis and 3D cell reconstruction were performed using the IMARIS 10.0.1 program (Oxford Instruments, Abingdon, UK). Initially, a surface was generated for GFAP^+^, IBA1^+^, or OLIG2^+^ immunosignals within their respective Z-stacks. Consistent surface detail and thresholding parameters were applied across stacks of the same staining parameters. For MAP2^+^-stained Z-stacks, the filament tool was applied to recreate the cytoskeletal protein, using the ‘Autopath (loops) no Soma and no Spine’ detection type and the ‘Multiscale Points’ option to enable the setting of variable filament diameters. The range of filament diameters remained consistent across all MAP2^+^-stained Z-Stacks, and the same threshold was applied throughout. The NHE1 immunosignal was replicated using the spots tool, with a uniform XY diameter across all stacks. Following the reconstruction of both green (GFAP^+^, IBA1^+^, OLIG2^+^, or MAP2^+^) and red (NHE1) signals, a filter was applied to remove all spots outside the surface or filament, retaining only the reconstructed NHE1 immunoreactive spots within the various reconstructed structures. Subsequently, the ‘Total Number of Spots’ was plotted for GFAP^+^, IBA1^+^, and OLIG2^+^ immunostaining, while the ‘Filament Length (Sum)’ was plotted for the MAP2^+^ immunostaining.

### 2.8. Colocalization Analysis

We employed the ImageJ (Version 1.53, NIH, Stapleton, NY, USA) colocalization tool JaCoP (Just Another Colocalization Plugin v.2.1.1) to quantify the extent of overlap between NOX2 immunostaining and cell-type markers. Stacked multichannel images were created from Z-stacks collected for each brain region and subsequently split by channel. The 8-bit channel images for NOX2 (imaged at 561 nm) and the cell-type marker (NeuN+ 488 nm) were subsequently analyzed by the JaCoP to determine M1 and M2 Mander’s Overlap Coefficients as described by the developers [20]. This generated the fraction of pixels positive for neuronal marker staining that also have NOX2^+^ immunostaining signal. All images were set to the same positive vs. negative immunostaining signal threshold during analysis.

### 2.9. Statistical Analysis

All experiments were conducted with impartial study design and analyses. Investigators were blinded to experimental groups until data analysis was complete whenever feasible. Power analyses were performed based on the mean and variability of data from our laboratory. N = 12 mice/group for behavioral tests, N = 6 mice/group for immunostaining, and MRI/DTI were sufficient to give us 80% power to detect 10% changes with 0.05 one-sided significance. Data were expressed as mean ± SEM and all data were tested for normal distribution using the Kolmogorov-Smirnov test. For comparing two conditions, a two-tailed Student’s *t*-test with 95% confidence was employed. For comparing three conditions or more, a one-way or two-way analysis of variance (ANOVA) analysis was used. Statistical significance was considered at a *p* value < 0.05 (Prism 10, GraphPad, San Diego, CA, USA). Non-normally distributed data were analyzed using a two-tailed unpaired Mann-Whitney U-test with a confidence level of 95% or other appropriate alternative tests according to the data. All data were included unless appropriate outlier analysis suggests otherwise.

## 3. Results

### 3.1. r-mTBI Mice Displayed Neurological Function Deficits in Both Acute and Chronic Phases

**Figure 1:**
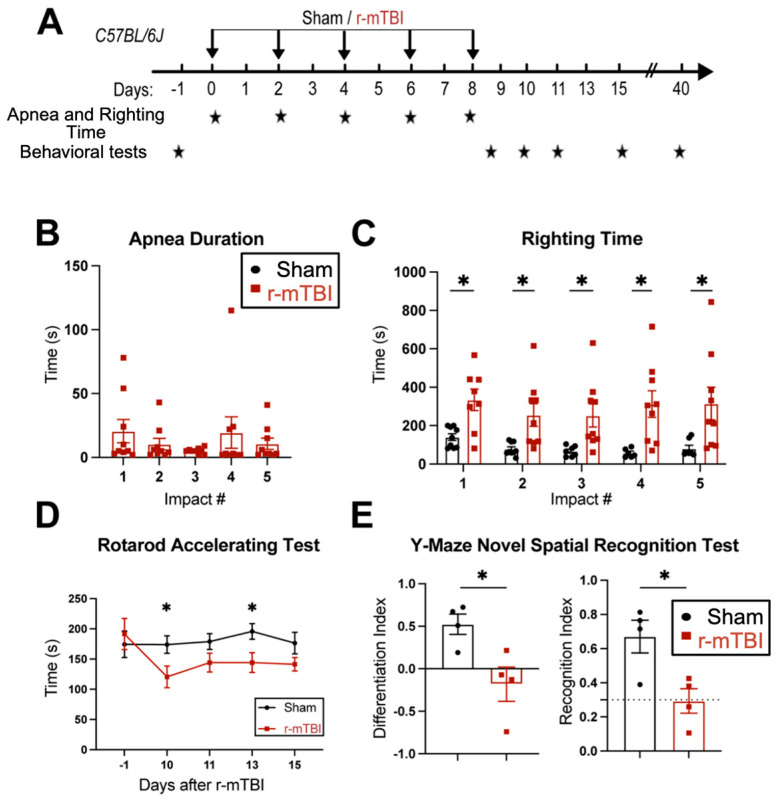
Experimental protocol and neurological function deficits induced by r-mTBI. (**A**). Experimental protocol: C57BL/6J mice (2–3 months old, male) were randomly assigned to sham control or r-mTBI groups (5 repetitive injuries with an inter-concussion interval of 48 h). (**B**,**C**). Apnea and righting times for sham and r-mTBI mice after each impact. N = 9. (**D**). Rotarod accelerating test in mice 1 day prior to r-mTBI induction at baseline and at 10–15 days post-first injury (dpi). N = 7 for sham group, N = 6 for r-mTBI group. (**E**). Y-maze novel spatial recognition test in a separate cohort of mice at 40 dpi. N = 4. Data are presented as mean ± SEM. * *p* < 0.05.

First, feasibility and features of the r-mTBI paradigm were characterized in an initial study. R-mTBIs were induced by repetitive injuries in C57BL/6J mice, with a total of five impacts on 0, 2, 4, 6, and 8 dpi, with an inter-concussion interval of 48 h (Figure 1A). Sham control mice underwent the same procedure without receiving the impacts. Following each impact, all r-mTBI animals exhibited a temporary cessation of breathing (apnea time) that was followed by an extended phase of immobility (righting time, Figure 1B). No significant differences were observed among apnea times detected between the injury days. Moreover, compared to the sham-operated mice, the r-mTBI mice demonstrated significantly higher righting times after all impacts (*p* < 0.05, Figure 1C), suggesting that a longer time for restoration of neurological function was required for r-mTBI mice [14]. Additionally, compared to the sham group, the r-mTBI mice displayed significantly poorer motor function in the rotarod accelerating test at 10 and 13 dpi (*p* < 0.05, Figure 1D). Behavioral testing of these animals during at 40 dpi revealed that r-mTBI mice demonstrated worsened performance in the y-maze novel spatial recognition test compared to sham mice. R-mTBI mice had significantly reduced differentiation indices (DI) and recognition indices (RI) compared to the sham mice (*p* < 0.05, Figure 1E), implying a poor spatial recognition memory function [15] (Figure 1E). No changes were detected on the spatial working memory test (y-maze spontaneous alternation test, Figure S3). In the open field test, the r-mTBI mice showed moderate hyperactivity compared to the sham mice (*p* = 0.08), but there were no statistically significant differences in locomotor activity or anxiety (Figure S3). Taken together, these findings indicate that the r-mTBI model triggered sustained motor and cognitive neurological function changes in both acute and chronic stages post-mTBI, which is consistent with previous reports [14]. These proof of concept experiments led us to conduct the subsequent experiments using the r-mTBI model with larger sample sizes.

### 3.2. r-mTBI Stimulates Robust Astrogliosis and Microgliosis in Cerebral Cortical, Hippocampal, and White Matter Tissues

**Figure 2:**
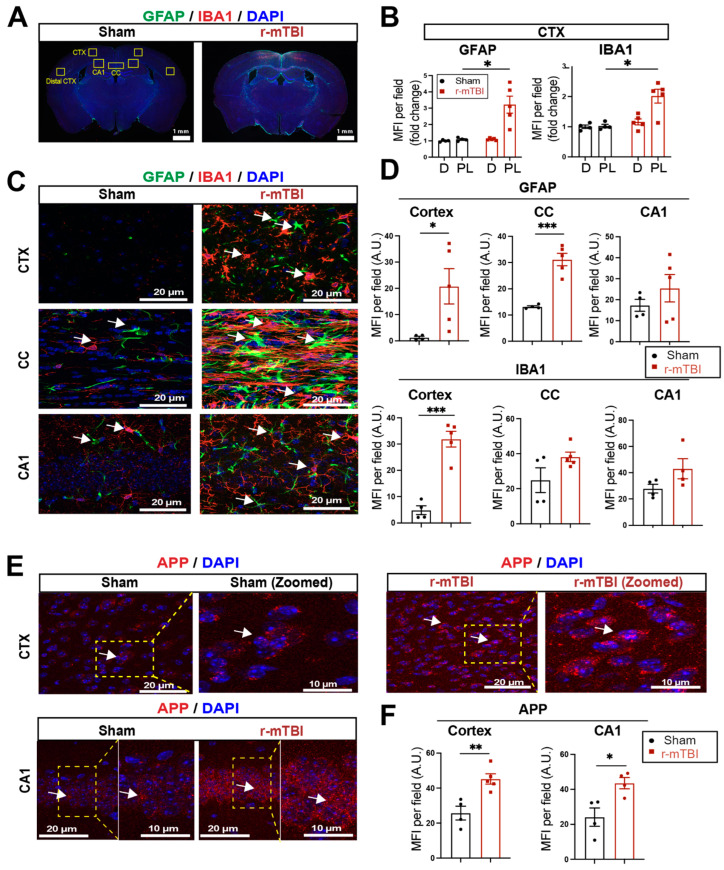
Robust astrogliosis and microgliosis induced by r-mTBI. (**A**,**B**). Representative low magnification (4×) immunostaining images of GFAP and IBA1 in the distal cortex (**D**) or peri-lesion cortex (PL) from sham or r-mTBI C57BL/6J brains at 15 dpi. GFAP^+^ and IBA1^+^ mean fluorescent intensity (MFI) fold change was quantified. (**C**,**D**). Representative confocal images (40×) and quantification of GFAP^+^ and IBA1^+^ MFI per field from cortex (CTX), corpus callosum (CC), and hippocampal CA1 regions at 15 dpi. (**E**,**F**). Representative confocal images and MFI quantification per field of these images for amyloid precursor protein (APP) in CTX and CA1 hippocampus at 15 dpi. N = 4 for sham group, N = 5 for r-mTBI group. Data are presented as mean ± SEM. Arrows: GFAP^+^, IBA1^+^ or APP^+^ cells. * *p* < 0.05, ** *p* < 0.01, *** *p* < 0.001.

We assessed levels of astrogliosis and microgliosis by immunostaining for the reactive astrocyte marker protein GFAP (glial fibrillary acidic protein) and the microglial marker protein IBA1 (ionized calcium-binding adaptor molecule 1) expression at 15 dpi. Compared to sham mice, r-mTBI mice displayed significant increases in immunofluorescence of the GFAP^+^ and IBA1^+^ protein especially in the bi-hemispheric peri-lesion areas (*p* < 0.05, Figure 2A,B). In contrast to the “resting” cellular morphology of both astrocytes and microglia in the sham brains, Figure 2C exemplifies the ameboid phenotypic morphology of microglia with larger cell bodies and fewer processes, and reactive astrocytes that displayed typical morphological changes such as hypertrophy and elongated cell processes, throughout the cortex (CTX), CC, and hippocampal CA1 regions of r-mTBI mice. This indicated pronounced activation of GFAP^+^ astrocytes and IBA1^+^ microglial cells in these brain areas at 15 dpi. Additionally, the mean fluorescent intensity (MFI) per field for GFAP^+^ astrocytes and IBA1^+^ microglia also significantly increased in the CTX and CC of these r-mTBI brains (*p* < 0.05, Figure 2C,D), suggesting certain brain regions are more susceptible to glial activation in response to r-mTBI. An examination of axonal damage (Figure 2E,F) showed an increased deposition of APP with significantly higher staining MFI in the CTX and hippocampal CA1 neurons of r-mTBI brains relative to sham brains (*p* < 0.05), suggesting r-mTBI induced axonal transport dysregulation [7,8]. Taken together, these data demonstrate the early occurrence of astrogliosis, microgliosis, and axonal damage in r-mTBI-induced pathogenesis development.

### 3.3. r-mTBI Stimulated NHE1 Protein Expression in Multiple Brain Cell Types

**Figure 3:**
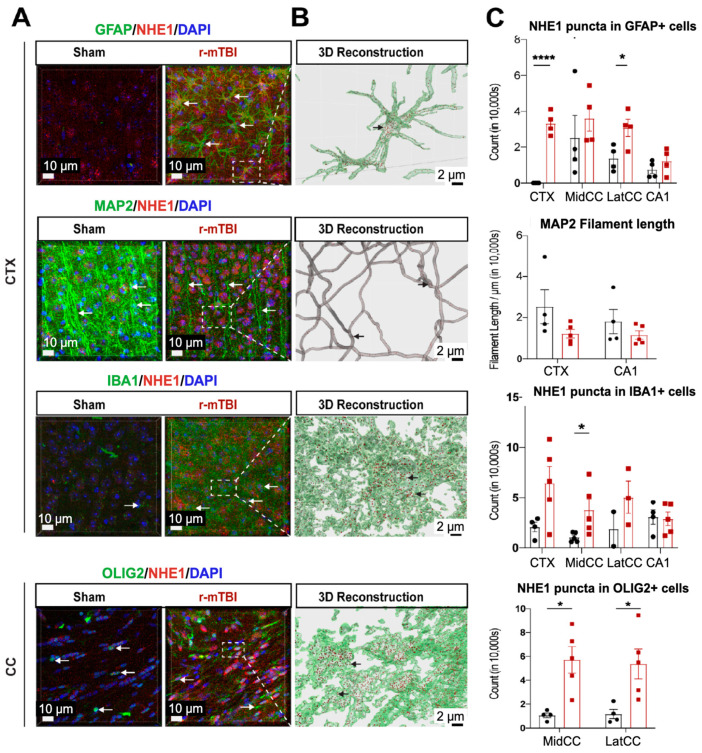
IMARIS 3D reconstruction in sham and r-mTBI brains. (**A**). Representative confocal (40×) double immunostaining images for NHE1 protein colocalizing with GFAP^+^ astrocytes, IBA1^+^ microglia, MAP2^+^ neurons, or OLIG2^+^ oligodendrocytes in CTX or CC from sham or r-mTBI brains at 15 dpi. (**B**). IMARIS 3D reconstruction images from the same dataset as in (**A**). (**C**). Quantification summary of NHE1^+^ puncta in GFAP^+^, IBA1^+^, and OLIG2^+^ cells, and MAP2^+^ filament lengths in sham or r-mTBI brains. Data are presented as mean ± SEM. N = 4 for sham group, N = 5 for r-mTBI group. Arrows: NHE1^+^ puncta in GFAP^+^, IBA1^+^, OLIG2^+^ cells and MAP2^+^ filaments. * *p* < 0.05, **** *p* < 0.0001.

NHE1 protein has been shown to be involved in astrocytic and microglial inflammatory responses after open-skull moderate-TBI and ischemic stroke mouse models [9,12]. Using immunofluorescence staining, we investigated whether NHE1 protein expression was altered in r-mTBI brains. Low levels of NHE1 protein expression were detected in GFAP^+^ astrocytes, IBA1^+^ microglia, and OLIG2^+^ oligodendrocytes throughout the CTX, CC, and CA1 regions in sham control brains (Figure 3A). In contrast, the r-mTBI brains showed significant increases in NHE1 protein immunoreactive signals in GFAP^+^ astrocytes, IBA1^+^ microglia, and OLIG2^+^ cells (arrows, Figure 3A) at 15 dpi. Imaris 3D reconstructed images show increased NHE1^+^ puncta in the soma and processes of GFAP^+^, IBA1^+^, and OLIG2^+^ cells of r-mTBI brains (Figure 3B). Increased NHE1 expression was most statistically significant in the GFAP^+^ astrocytes in the CTX and CC regions of r-mTBI brains (*p* < 0.0001 and *p* < 0.05, respectively; Figure 3C), in the OLIG2^+^ cells in the CC regions (both the middle and lateral, *p* < 0.05; Figure 3C), and in IBA1^+^ microglia (in the middle CC region, *p* < 0.05; Figure 3C). While we did not detect changes in NHE1 protein expression in MAP2^+^ filaments between sham and r-mTBI brains, we found a decrease in MAP2^+^ filament lengths in the CTX and CA1 regions of the r-mTBI brains that did not reach statistical significance (*p* > 0.05; Figure 3C). Taken together, these data suggest that r-mTBI increased expression of NHE1 protein in various cell types in multiple brain regions. These findings motivated us to investigate whether the blockade of NHE1 protein would alter brain cell homeostasis or r-mTBI-mediated brain damage.

### 3.4. Post-Injury Administration of Selective NHE1 Protein Inhibitor HOE642 Significantly Improved Neurological Behavioral Functions Post-r-mTBI

**Figure 4:**
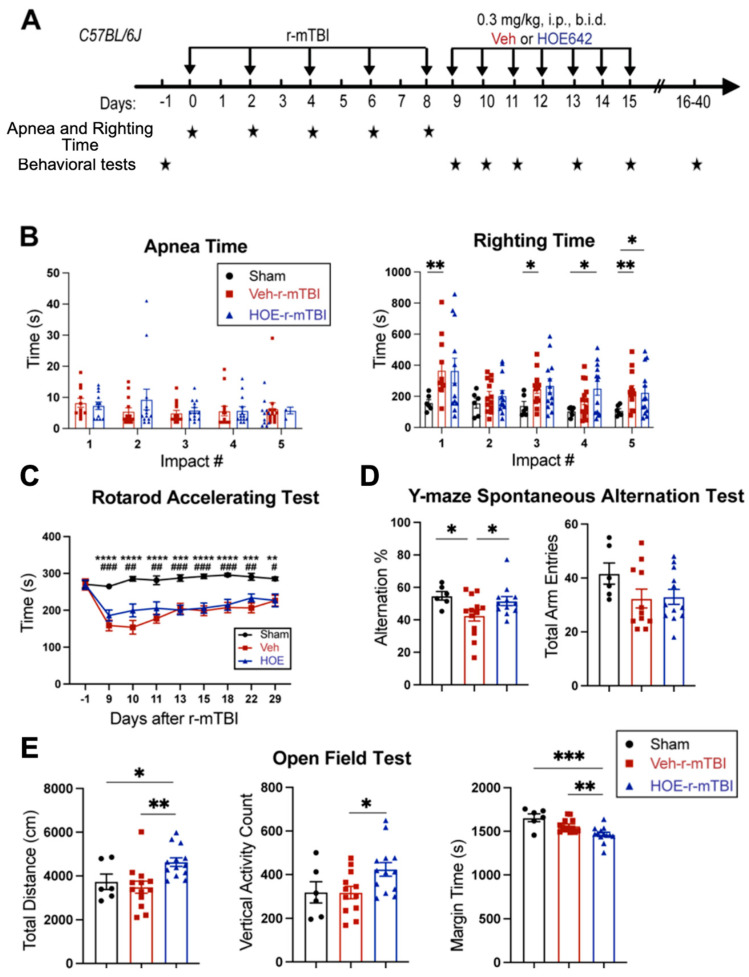
Efficacy of NHE1 inhibitor HOE642 on improving behavioral performance in r-mTBI mice. (**A**). Experimental protocol. Veh (DMSO) or HOE642 (0.15 mg/kg body weight/day, b.i.d., 8 h apart) was administered from 9–15 dpi in C57BL/6J mice (2–3 months old, male). (**B**). Apnea and righting times. (**C**). Rotarod accelerating test at baseline (1 day prior to r-mTBI or sham induction) and at 9–29 dpi. **|**Sham vs. Veh-r-mTBI ** *p* < 0.05, *** *p* < 0.001, **** *p* < 0.0001; **|**Sham vs. HOE-r-mTBI # *p* < 0.05, ## *p* < 0.01, ### *p* < 0.001. (**D**). Y-maze spontaneous alternation test conducted at 32 dpi from the same cohort of mice as in (**D**). (**E**). Open field test conducted at 30 dpi from the same cohort of mice as in (**D**). Data are presented as mean ± SEM. N = 6 for sham group, N = 13 for Veh-treated group, N = 13 for HOE-treated group. * *p* < 0.05, ** *p* < 0.01, *** *p* < 0.001.

We examined whether a delayed administration of NHE1 protein inhibitor HOE642 could attenuate neurological deficits in r-mTBI mice. Figure 4A illustrates the regimen of administration of Veh (DMSO) or HOE642 (0.3 mg/kg body weight/day, b.i.d., 8 h apart) from 9–15 dpi post-sham or r-mTBI. In general, sham mice had significantly lower righting times than the r-mTBI mice (Veh- and HOE-cohort) after impact #1 (*p* < 0.01) and impacts #4–5 (*p* < 0.05), suggesting that r-mTBI mice required a longer time for neurological restoration when compared to sham mice [14]. Similar apnea and righting times were detected in the Veh- and HOE-cohort mice prior to their treatment, suggesting similar initial neurological damage in the two groups (Figure 4B). Moreover, compared to sham animals, all r-mTBI mice (both Veh- and HOE-treated mice) exhibited a significantly poorer performance in the rotarod accelerating test at 9–29 dpi (*p* < 0.01; Figure 4C). Compared to the Veh-treated r-mTBI mice, the HOE-treated r-mTBI mice showed improved motor performance with higher latency on the rotating rods at 9, 10, 11, and 22 dpi (Figure 4C, *p* = 0.1). The Veh-treated r-mTBI mice also displayed sustained working memory deficits with a significant decrease in arm alternations (with similar arm entries) in the y-maze spontaneous alternation test at the chronic phase of 32 dpi, compared to the sham mice (*p* < 0.05; Figure 4D), which was almost fully attenuated in the HOE-treated r-mTBI group (Figure 4D, *p* < 0.05). At 30 dpi, the Veh-treated mice exhibited similar locomotor activity in the open field test as the sham control mice (Figure 4E). Interestingly, the HOE-treated r-mTBI mice showed significantly stimulated locomotor functions reflected by increased total travel distance and vertical activity counts (*p* < 0.05; Figure 4E). These HOE-treated r-mTBI mice concurrently displayed reduced anxiety, indicated by significantly decreased margin time than the Veh-treated r-mTBI mice (*p* < 0.01; Figure 4E). However, no significant differences in the index ratios (DI, *p* = 0.3; and RI, *p* = 0.2) were observed between the Veh-treated and HOE-treated r-mTBI mice when assessed with the y-maze novel spatial recognition test at 39 dpi, suggesting negligible differences in spatial reference memory between the two groups (Figure S4). Taken together, these findings demonstrated that post-r-mTBI pharmacological blockade of the NHE1 protein with HOE642 significantly improved both locomotor and cognitive neurological functions in the subacute and chronic phase of r-mTBI.

### 3.5. Pharmacological Inhibition of NHE1 with HOE642 Reduced Gliosis and Oxidative Damage over Broad Brain Regions after r-mTBI

**Figure 5:**
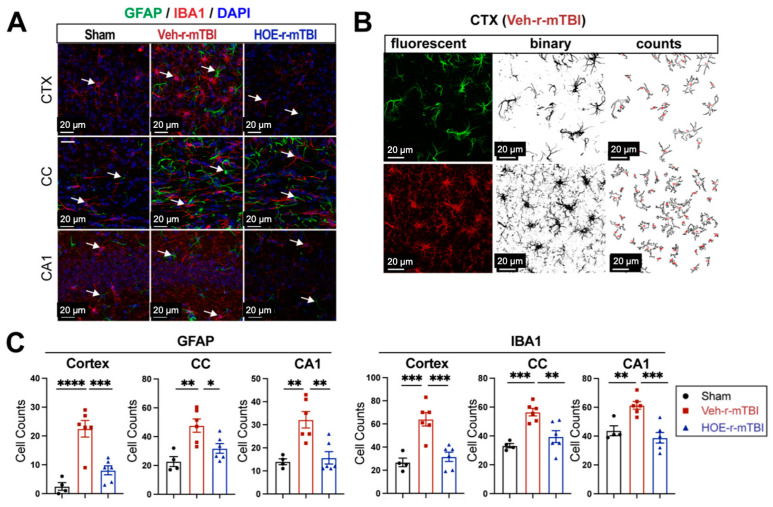
Efficacy of pharmacological blocking of NHE1 protein on reducing gliosis and oxidative damage after r-mTBI. (**A**). Representative confocal immunofluorescent images (40×) of GFAP^+^ and IBA1^+^ cells at 60 dpi in the CTX, CC, and hippocampal CA1 regions (**B**,**C**). Representative confocal immunofluorescent and binary images (40×) used for the unbiased semi-automatic quantification of GFAP^+^ and IBA1^+^ cell counts at 60 dpi. N = 4 for sham group, N = 6 for Veh-treated r-mTBI group, N = 6 for HOE-treated r-mTBI group. Data are presented as mean ± SEM. Arrows: GFAP^+^ or IBA1^+^ cells. * *p* < 0.05, ** *p* < 0.01, *** *p* < 0.001, **** *p* < 0.0001.

To evaluate whether delayed pharmacological blockade of the NHE1 protein would impact levels of astrogliosis and microgliosis following r-mTBI, we conducted immunostaining for GFAP^+^ and IBA1^+^ expression in the CTX, CC, and CA1 regions in both Veh- and HOE-treated r-mTBI mice at 60 dpi. Compared to sham-operated mice, the Veh-treated r-mTBI mice exhibited amoeboid cell morphologies for both GFAP^+^ and IBA1^+^ cells (Figure 5A), akin to those observed in the r-mTBI cohort at 15 dpi (Figure 2C). Significant increases in GFAP^+^ and IBA1^+^ cell counts in the three brain regions were also identified through unbiased semi-automatic quantification of cell counts using binary images in ImageJ software (Figure 5B,C, *p* < 0.01), indicating robust activation of both reactive astrocytes and microglia. In contrast, the HOE-treated r-mTBI brains displayed similar cell morphologies to the resting phenotypes observed in sham brains at 15 dpi (Figure 5A and Figure 2C), along with significantly reduced cell counts of both GFAP^+^ and IBA1^+^ cells throughout all three brain regions (Figure 5C, *p* < 0.05).

**Figure 6:**
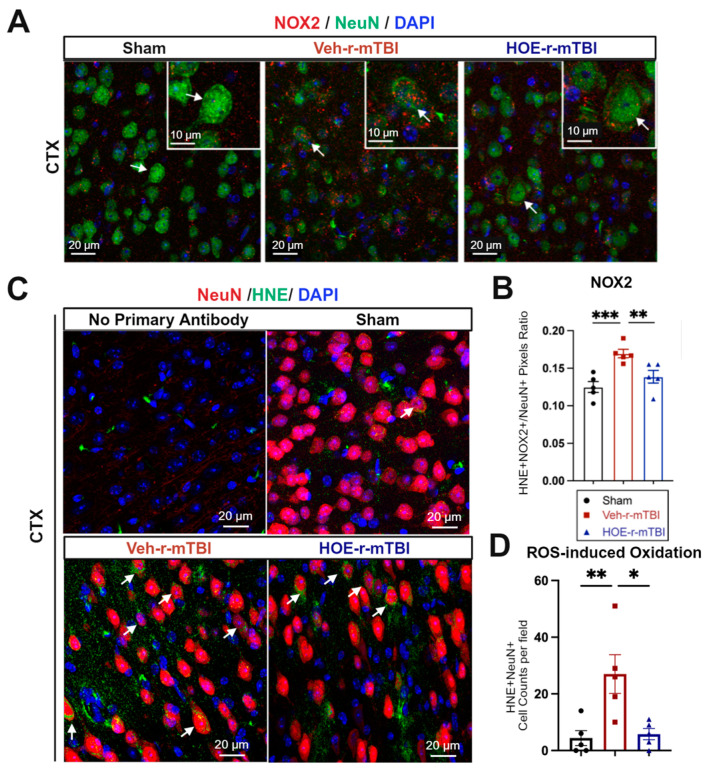
Efficacy of pharmacological blocking of NHE1 protein on reducing NOX2 activation and oxidative damage after r-mTBI. (**A,B**). Representative confocal immunofluorescent images (40×) and quantification of NOX2 expression in NeuN+ cells at 60 dpi. Insert: 2× zoom from the respective image, with an arrow indicating NOX2+NeuN+ cells. (**C**,**D**). Representative confocal immunofluorescent images (40×) and quantification of HNE in NeuN+ cells at 60 dpi. Arrow: HNE+NeuN+ cells. N = 5 for sham group, N = 5 for Veh-treated r-mTBI group, N = 5 for HOE-treated r-mTBI group. Data are presented as mean ± SEM. * *p* < 0.05, ** *p* < 0.01, *** *p* < 0.001.

In evaluating oxidative stress-induced changes, immunostaining of phosphor-p47-phox (p-p47), a crucial active subunit in the NOX2 complex activation [21], was performed in r-mTBI brains at 60 dpi. We observed that the Veh-treated r-mTBI brains exhibited visually higher p-p47 NOX2 expression within NeuN^+^ neurons in the CTX, reflected by a significantly higher overlap percentage when compared to sham brains (arrows, Figure 6A,B, *p* < 0.001). Conversely, HOE-treated r-mTBI mice displayed lower p-p47 NOX expression within NeuN^+^ neurons in the CTX, appearing more akin to sham tissue at 60 dpi (arrows, Figure 6A). Additionally, HOE-treated r-mTBI brains exhibited a significant decrease in the overlap percentage of p-p47 NOX2 expression within NeuN^+^ cortical neurons (arrows, Figure 6A,B, *p* < 0.01) when compared to Veh-treated r-mTBI mice. To determine whether r-mTBI triggers pathological oxidation downstream of ROS production, we conducted immunofluorescence staining for 4-hydroxy-2-nonenal (HNE). HNE is formed by ROS peroxidation of cell membranes and is a well characterized marker of oxidative stress [22,23]. Sham animal cortices exhibited minimal HNE staining, while r-mTBI-Veh brains showed a 3-fold relative increase in the mean number of HNE+ NeuN+ neurons per field (Figure 6C,D, *p* <0.01). Treatment with HOE 642 significantly reduced the formation of HNE in cortical neurons after r-mTBI (*p* < 0.05).

In summary, these findings demonstrate that delayed pharmacological blockade of the NHE1 protein reduces r-mTBI-mediated astrogliosis and microgliosis, as well as oxidative stress responses in neurons.

### 3.6. Pharmacological Inhibition of NHE1 with Inhibitor HOE642 Attenuated Axonal and White Matter Damage after r-mTBI

**Figure 7:**
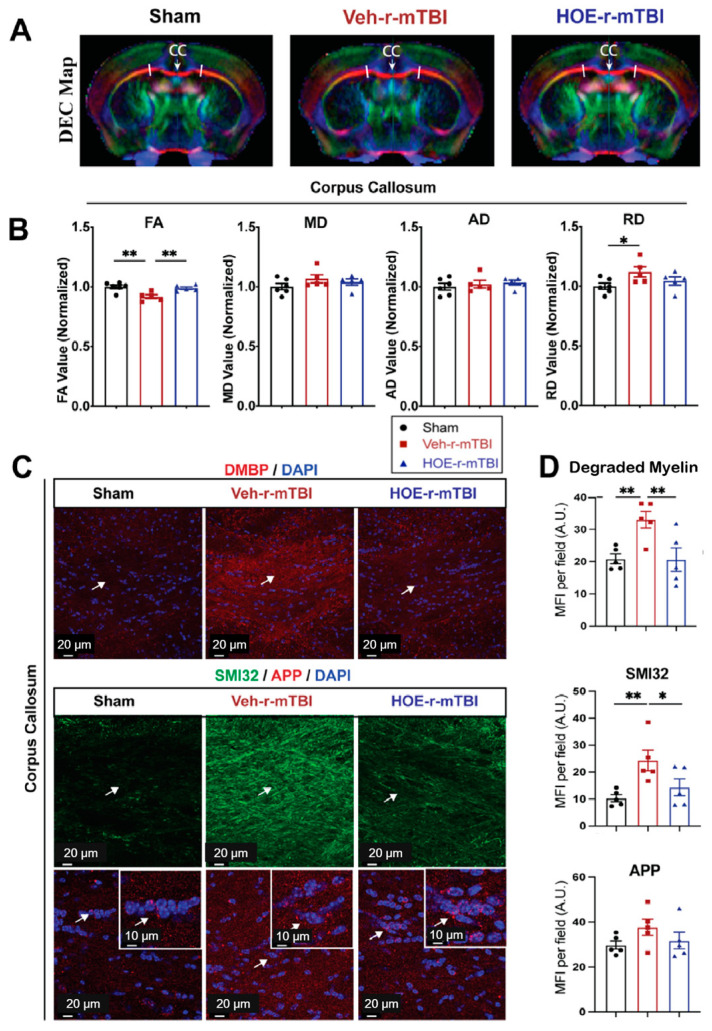
Changes in r-mTBI-induced axonal damage detected by MRI Diffusion Tensor Imaging (DTI) and immunostaining. (**A**). Representative DTI directionally encoded color (DEC) map of ex vivo brains from sham, Veh-treated and HOE-treated r-mTBI mice at 60 dpi. (**B**). Analysis of normalized fractional anisotropy (FA), mean diffusivity (MD), axial diffusivity (AD), and radial diffusivity (RD) values in the corpus callosum (CC) from the same cohort of mice in A. N = 6 for sham group, N = 5 for Veh-treated group, N = 5 HOE-treated group. (**C**,**D**). Representative confocal immunofluorescent images (40×) and MFI quantification of degraded myelin basic protein (DMBP), SMI32, and APP at 60 dpi in the CC from the same cohort of mice as in A. Insert: 2× zoom of the respective image with arrows indicating the areas of interest. Data are presented as mean ± SEM. N = 5 for sham group, N = 5 for Veh-treated group, N = 5 HOE-treated group. Arrows: CC location, DMBP, SMI32, and APP expression. * *p* < 0.05, ** *p* < 0.01.

To gain a deeper understanding of r-mTBI-mediated white matter damage, MRI DTI studies were conducted in ex vivo brains of sham, Veh-treated, and HOE-treated r-mTBI mice at 60 dpi (Figure 7A). The representative directionally encoded color (DEC) maps showed no noticeable lesions in the white matter tracts (arrow, Figure 7A) among the three groups. The DTI metric FA is the most widely used among DTI indices and is often equated with white matter integrity [18]. FA provides a measurement that characterizes the overall directionality of water diffusion within tissues, such as the white matter tracts in the brain [24]. Lower values indicate a loss of integrity that can occur from conditions that cause axonal damage or demyelination [24]. Within the CC, Veh-treated r-mTBI mice exhibited a significant reduction in FA values (normalized to sham control animals) (Figure 7B, *p* < 0.01).

In contrast, the HOE-treated r-mTBI mice demonstrated preserved FA values (normalized to sham control animals) (Figure 7B, *p* < 0.01) when compared to Veh-treated r-mTBI mice. RD is a DTI measurement that characterizes the diffusion of water molecules perpendicular to the principal axis of diffusion within tissues [24]. In contrast to FA, RD values tend to increase in conditions such as demyelination or changes in axonal density [24]. Interestingly, the DTI metric RD (normalized to sham) showed a significant increase (Figure 7B, *p* < 0.05) in the Veh-treated r-mTBI cohort when compared to sham animals. Other normalized DTI metrics, such as MD or AD, showed no significant changes across the three groups. Within the hippocampal CA1 regions and external capsule, no significant differences were detected in normalized FA, AD, MD, or RD values between the three groups (Figure S5). Within the internal capsule, no significant differences were detected in normalized FA, AD, or MD values between the three groups (Figure S5). However, compared to sham mice, the HOE-treated r-mTBI mice had a statistically significant increase in the DTI metric RD (normalized to sham, Figure S5).

To further support our DTI findings, we conducted immunostaining for damaged myelin with the degraded myelin basic protein (DMBP) antibody [25], and for axonal damage with SMI32 (non-phosphorylated neurofilament) and APP in the CC (Figure 7C) [16,26]. When compared to sham mice, Veh-treated r-mTBI mice had a significantly higher MFI of DMBP and SMI32 expression (arrows, Figure 7C,D, *p* < 0.01) within the CC. In contrast, the HOE-treated r-mTBI mice exhibited a significant decrease in the MFI for both DMBP and SMI32 compared to the Veh-treated r-mTBI mice in the CC (arrows, Figure 7C,D, *p* < 0.01 and *p* <0.05, respectively). However, no differences were detected in the MFI of APP accumulation among all three groups (arrows, Figure 7C,D). Taken together, our unbiased MRI DTI findings, in addition to our immunostaining data, demonstrate that pharmacological inhibition of the NHE1 protein reduced axonal damage and preserved white matter integrity in the CC following r-mTBI.

### 3.7. Selective Deletion of Microglial Nhe1 in Cx3cr1^CreER+/−^;Nhe1^f/f^ Mice Reduced Astrogliosis after r-mTBI

**Figure 8:**
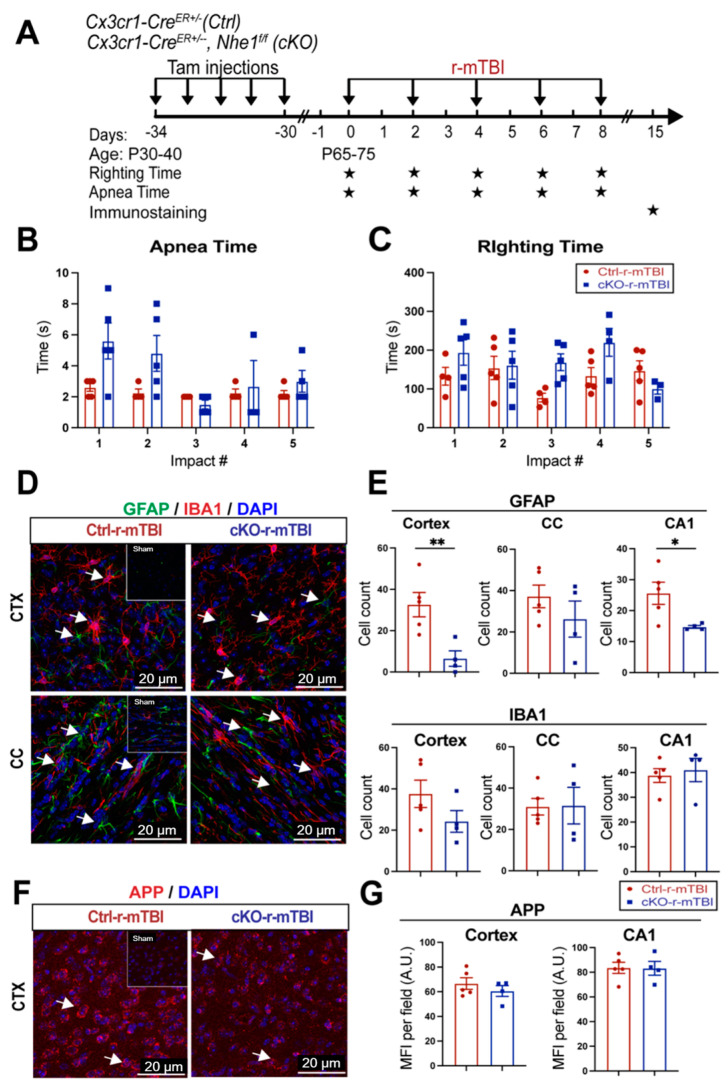
Selective deletion of microglial *Nhe1* in *Cx3cr1^CreER+/−^;Nhe1^f/f^* mice reduces astrogliosis. (**A**). Experimental protocol: Tamoxifen (Tam, 75 mg/kg body weight, 20 mg/mL in corn oil, i.p.) was administered in Ctrl (*Cx3cr1^CreER+/−^* mice) and *Cx3cr1^CreER+/−^*; *Nhe1^f/f^* (cKO mice) daily for 5 consecutive days. A 30-day waiting period was given for complete clearance of Tam and peripheral Cx3cr1^+^ monocyte turnover. Repetitive injuries of a total of 5 impacts with an inter-concussion interval of 48 h were induced in both Ctrl and cKO mice. (**B**,**C**). Apnea and righting times. (**D**). Representative immunofluorescent images (40×) of GFAP^+^ and IBA1^+^ cells at 15 dpi. Insert: Sham mice from separate cohort perfused at 15 dpi. (**E**). Unbiased semi-automatic quantification of GFAP^+^ and IBA1^+^ cell counts from the CTX, CC, and hippocampal CA1 regions at 15 dpi. (**F**). Representative immunofluorescent images (40×) of APP staining in the CTX at 15 dpi. Insert: Sham mice from separate cohort perfused at 15 dpi. (**G**). MFI quantification of APP expression in the CTX and hippocampal CA1 regions. Arrows: GFAP^+^, IBA1^+^ or APP expression. Data are presented as mean ± SEM. N = 5 for Ctrl group, N = 4 for cKO groups. * *p* < 0.05, ** *p* < 0.01.

Following TBI, pro-inflammatory microglia can become activated, leading to the release of pro-inflammatory cytokines and ROS that exacerbate neuronal injury [9]. In our previous study, microglia-specific *Nhe1* cKO mice displayed an increase in the anti-inflammatory, restorative microglia phenotype, which subsequently led to decreased GFAP^+^ and IBA1^+^ cell counts and improved white matter remyelination and oligodendrogenesis following an open-skull, moderate-TBI model [9]. We investigated here whether *Nhe1* cKO mice show resilience to r-mTBI-induced damage. r-mTBI triggered similar apnea and righting times in the Ctrl and cKO mice (Figure 8A,B). Interestingly, the microglia-specific *Nhe1* cKO mice displayed significantly reduced GFAP^+^ cell counts throughout the CTX and CA1 regions compared to Ctrl mice at 15 dpi after r-mTBI (Figure 8D,E, *p* < 0.01 and *p* < 0.05, respectively). However, while overall cell counts of IBA1^+^ cells appeared to be reduced in the CTX of *Nhe1* cKO mice compared to Ctrl mice after r-mTBI (Figure 8D,E), this difference lacked statistical significance. Immunostaining for APP accumulation did not show any significant differences between these two groups (Figure 8F,G). These results show that specific deletion of *Nhe1* in microglial cells has profound effects on attenuating astrogliosis after repetitive injuries; however, it was not effective in reducing the levels of activated microglia or APP accumulation from damaged axons.

## 4. Discussion

### 4.1. Clinical Significance of mTBI and Pathogenesis of White Matter Damage

It has been extensively documented that DAI is a characteristic pathology following mTBIs [7,27]. DAI not only manifests following mTBIs but across all spectrums of TBI severities [7,28]. This is mainly attributed to the susceptibility of axonal projections in the white matter tracts to shear after experiencing rapid acceleration or deceleration forces, causing microscopic damage to the brain’s axons [7,28]. Key features of DAI encompass axonal tearing, mitochondrial swelling, and the formation of axonal bulbs, subsequently leading to disruptions in neuronal transport [4,7,28] and protein accumulation, often evidenced as the accumulation of APP [7,8]. APP accumulation has been consistently observed in cases of mild trauma and occurs quite rapidly after the inciting event, as evidenced by postmortem studies conducted on individuals who have experienced mTBIs and died from unrelated causes [29]. In our study, we observed a significantly higher MFI of APP accumulation in both the CTX and hippocampal CA1 regions at 15 dpi (Figure 2E). APP is transported via fast anterograde axon transport along microtubules and serves as a sensitive marker of acute axonal disruption in white matter during brain damage [7,8,30]. At 60 dpi, we observed no significant difference between sham, Veh-treated, and HOE-treated r-mTBI mice for APP accumulation in the CC. This prompted us to stain for SMI32, a well-validated marker that stains non-phosphorylated neurofilaments for detecting axonal damage [16,26]. Under physiological conditions, neurofilaments are transported through slow axonal transport [31]. In pathological conditions that result in disrupted transport or abnormal phosphorylation, they accumulate within affected axons [32], contributing to pathological processes. In the CC at 60 dpi, we observed a significantly increased MFI of SMI32 in the Veh-treated r-mTBI mice compared to sham (Figure 7C,D) and a significant decrease in SMI32 MFI in the HOE-treated r-mTBI mice compared to Veh-treated mice (Figure 7C,D). Taken together, these results indicate that HOE administration did reduce r-mTBI-induced DAI by the chronic timepoint of 60 dpi.

Diagnosing mTBI-induced DAI in the clinical setting remains challenging, as traditional imaging methods, such as computed tomography (CT), are insufficient for detecting the specific microscopic changes found throughout the brain. However, advanced imaging modalities such as MRI DTI show more promise for diagnosing axonal injuries and detecting changes in white matter integrity [33,34]. The DEC color map illustrates the orientation of water diffusion, facilitating the detection of the alignment of white matter pathways [33]. Furthermore, DTI enables the construction of tract bundles from ROIs and analysis of their diffusion metrics [33,34]. The primary diffusion metric in DTI, FA, serves as an indicator of white matter tract integrity [33,34,35]. Clinical research studies on mTBI patients with chronic symptoms revealed that damage to axons in white matter pathways commonly results in reduced FA values [36,37]. In our study, we observed significant reductions in FA in the Veh-treated r-mTBI mice in the CC at 60 dpi, compared to sham mice (Figure 7A,B), while HOE-treated r-mTBI mice displayed restored FA values (Figure 7A,B). Additionally, compared to sham mice, Veh-treated mice also exhibited a significant increase in RD (Figure 7A,B), suggesting increased demyelination from r-mTBI induction in these animals [24]. These findings imply reduced white matter integrity in the Veh-treated r-mTBI mice compared to HOE-treated mice. In addition, within the internal capsule, when compared to sham animals, HOE-treated r-mTBI animals were observed to have higher RD values as well (Figure S5). This also indicates that HOE-treated animals had higher levels of demyelination when compared to sham, as HOE-treated animals also underwent r-mTBI [24]. Our findings were further validated by a significantly higher MFI of DMBP (Figure 7C), a marker for abnormal oligodendrocyte processes found within demyelinating areas [25], in the Veh-treated mice. Collectively, our findings suggest that post-r-mTBI administration of the NHE1 inhibitor, HOE642, reduces axonal damage and improves white matter integrity. Moreover, these findings suggest promising future applications of MRI DTI in facilitating clinical diagnoses for patients with mTBIs.

### 4.2. TBI-Induced Brain pH Dysregulation and NHE1 Upregulation

Findings from recent decades have firmly established post-traumatic cerebral acidosis as a hallmark of secondary injury following TBI [38,39]. Brain acidosis, characterized by an increase in the brain lactate/pyruvate ratio, has been shown to be associated with worsened outcomes in acute severe TBI patients [40]. pH dysregulation, both intracellular and extracellular, have been detected after moderate-TBIs [41]. While the heterogeneity of brain injuries may influence the precise mechanisms and clinical implications of this process, it is widely acknowledged that increased extracellular levels of pCO2, protons, and lactate correspond to slower recovery [42], as well as an elevated risk of poorer outcomes and mortality [43,44,45]. This consensus is evident in the rigorous clinical regulation of ventilation and pO2/pCO2 pressures after severe brain injury [46]. Additionally, extracellular acidic conditions following TBI are believed to contribute to the accumulation of tau and amyloid-β peptide aggregates observed in chronic traumatic encephalopathy [47,48]. This evidence indicates that mitigating the pathological H^+^ homeostasis dysregulation is a potential approach for modulating post-mTBI pathogenesis.

At the cellular level, the decrease in intracellular and extracellular pH is associated with distorted metabolism and upregulated production of ROS, which can damage cell membranes and impair critical cellular functions [41]. Numerous mediators of this pathogenesis are sensitive to pH, such as the activation of NOX activity and the production of superoxide ROS production under acidic conditions [11]. In our study, we observed consecutive mTBIs-induced increases in NHE1 immunoreactivity in cortical neurons, as well as in activated astrocytes, oligodendrocytes, and microglia in the CC (Figure 3C). This upregulation of NHE1 may stimulate H^+^ efflux in response to elevated intracellular proton and lactate levels after brain injury. Consequently, the activation of proton extrusion proteins like NHE1 is crucial for maintaining the optimal pH_i_ for sustained NOX activation, leading to ROS production and oxidative damage following brain injuries [4,9], and allowing NOX-driven inflammation and cell damage to persist [11,16]. This was supported by our observation of increased neuronal expression of p-p47, the initiating component of the NOX2 complex activation, in the Veh-treated r-mTBI animals (Figure 6A,B). Other proteins involved in H^+^ efflux, such as the voltage-gated H^+^ channel (Hv1) [49,50] and the ATPase H^+^ [51], maintain intracellular pH_i_ in microglial cells through proton extrusion, similar to the NHE1 protein after brain injury [9]. Interestingly, previous research also showed that Hv1 channel activation in microglia cells is required for NOX-mediated oxidative damage, and inhibition of this specific channel in microglia reduced NOX activation and neuronal cell death as early as 24 h after ischemic stroke [50]. Collectively, our study demonstrates that pharmacological inhibition of NHE1 protein activity prevents the activation of the p-p47 NOX2 subunit in neurons and mitigates oxidative damages induced by r-mTBI.

### 4.3. Antioxidant Effects of NHE1 Protein Inhibitor

**Figure 9:**
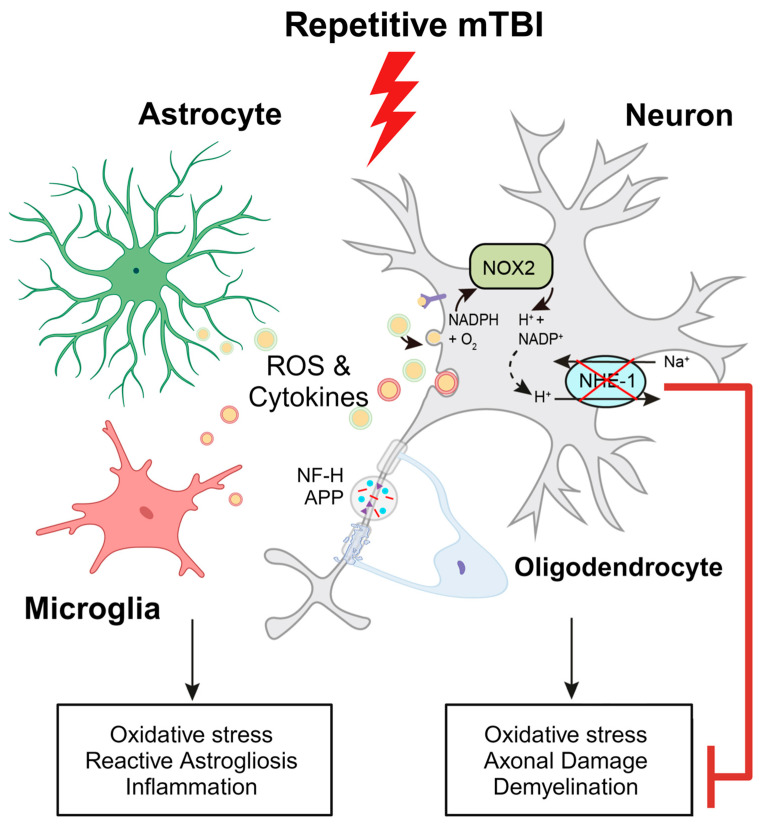
Schematic illustration of r-mTBI-induced neuronal damage associated with the activation of NOX2 and NHE1. R-mTBI induces upregulation of the NHE1 protein in neurons and various cell types. The interaction between NHE1 with NOX2 leads to oxidative stress-induced neuroinflammation and white matter damage following r-mTBI. Pharmacological inhibition of NHE1 reduces gliosis, oxidative stress, and axonal injury while improving locomotor and cognitive functional recovery. Overall, these findings suggest that blocking NHE1 activity inhibits reactive astrocyte and inflammatory microglia responses, promotes neuroprotection, and enhances white matter integrity, thereby expediting neurological function recovery following r-mTBI. Illustration created with Biorender.com and Adobe Illustrator software 14.4 (Adobe Inc., Mountain View, CA, USA). Abbreviations: APP, amyloid precursor protein; NFL-H, neurofilament heavy chain; NHE1, sodium-hydrogen exchanger 1; NOX2, NADPH oxidase; ROS, reactive oxygen species.

HOE642 (Cariporide) is a selective and potent inhibitor of the NHE1 protein, effectively blocking NHE1-mediated H^+^ extrusion (along with blocking Na^+^ influx). NHE1 inhibition acidifies resting pH_i_ from approximately ~7.0 to ~6.8 in neurons, akin to reducing the extracellular pH (pH_e_) from 7.2 to 7.0 [11]. HOE642 has been shown to exert antioxidant benefits: in vitro administration of HOE642 nearly abolished free radical formation and neuronal death after NMDA-induced excitotoxicity [11] or oxygen-glucose deprivation/reoxygenation (OGD/REOX) via NOX2 complex suppression [52]. Similarly, in vivo administration of HOE642 demonstrated potency in reducing NOX activation and superoxide production across various diseased models, including ischemic stroke [53] and chronic cerebral hypoperfusion [54]. Our current findings further demonstrate NHE1 protein activation and its relationship to activation of the cytosolic NOX2 subunit and ROS-mediated HNE formation in r-mTBI brains (Figure 9). We also revealed that post-r-mTBI administration of the NHE1 inhibitor HOE642 for one week after five CCI-induced r-mTBIs significantly reduced p-p47 NOX2 expression and HNE production in cortical neurons (Figure 6). These data indicate the crucial roles of NHE1-mediated pH_i_ regulation in oxidative damage and neuroinflammation following r-mTBI. The observed antioxidant effects of HOE642 likely led to the reduced axonal and white matter damage with diminished accumulation of SMI32 and DMBP (Figure 7C,D), alongside increased FA values in the CC detected with MRI DTI (Figure 7A,B). This was accompanied by reduced microgliosis and astrogliosis (Figure 5A,C), as well as improved locomotor and cognitive functional recovery (Figure 4C–E) in the HOE642-treated mice post-r-mTBI. Treatment options for axonal damage induced by mTBIs are currently limited, as existing therapies primarily target addressing presenting signs and symptoms [4]. Our novel data indicate that targeting the NHE1 protein with the pharmacological inhibitor HOE642 could provide antioxidant benefits and therapeutic potentials to mTBI patients.

Additional mechanisms of NHE1-mediated pH_i_ homeostasis include maintaining balanced aerobic glycolysis and oxidative phosphorylation (OXPHOS) [16,55,56]. Metabolic reprogramming from OXPHOS to glycolysis is associated with neurodegenerative phenotypes of microglia, while restoration of OXPHOS activity, with a reversal of glycolytic metabolism, mitigated the pathological changes associated with Alzheimer’s disease [57]. Although the energy demand of astrocytes is predominantly met by glycolysis, their OXPHOS activity is required to provide crucial nutrient support to neurons by degrading fatty acids and maintaining lipid homeostasis [58]. Defects in astrocytic OXPHOS induce lipid accumulation and reactive astrogliosis, subsequently suppressing oligodendrocyte-mediated myelin generation [58]. Whether HOE642-mediated neuroprotective effects are mediated by metabolic reprogramming in neurons and glial cells remains to be further elucidated.

To our knowledge, HOE642 remains one of the best NHE1 inhibitors with high selectivity, high potency (with IC50 as low as 4.5 nM), and minimal reported side effects [59,60]. Observed clinical side effects are less frequent compared to amiloride, the first synthesized NHE1 inhibitor, and its derivatives, which target both NHE1 and NHE2 isoforms and thus often present off-target effects [61]. HOE642, as the next generation of NHE1 inhibitors, replaces the pyrazine core in amiloride with a phenyl ring and presents both higher selectivity and sensitivity for the NHE1 isoform [61]. Because NHE1 is quiescent in physiological conditions and is activated only during acidosis or cell shrinkage [59], pharmacological application of NHE1 inhibitors likely has minimal effects under physiological conditions. However, the specific effects of HOE642 in naïve or sham-operated mice warrant additional characterizations.

### 4.4. Lack of Reduction of Microgliosis in Microglial-Specific Nhe1 cKO Mice after r-mTBI

Neuroinflammation and activation of reactive astrocytes and microglia are pathological hallmarks in concussion TBI models [62,63]. Activation of oxidative stress and proinflammatory signaling pathways, such as nuclear factor κ-light chain-enhancer (NF-κB) and mitogen-activated protein kinase (MAPK), have been reported to play a role in these events [64,65]. Compared to WT Ctrl, *Nhe1* cKO mice showed a pronounced reduction in GFAP^+^ astrocyte cell counts but did not exhibit any changes in IBA1^+^ microglia or APP accumulation in any of the three brain regions at 15 dpi following r-mTBI (Figure 8). However, our recent report found a profound reduction in microglial activation in *Nhe1* cKO mice in an open-skull CCI contusion model [9]. The causes of these different outcomes are not apparent. Possible reasons could be attributed to small sample size (n = 4–5) and/or a less significant role of microglial NHE1 protein in r-mTBI pathogenesis (milder injury, chronic timepoint for collection), compared to other cell types (neuronal, astrocytes, etc.). Moreover, in addition to cell count, a more detailed analysis of microglial activity should be further conducted.

This study has additional limitations. While the selected sample sizes were sufficient to detect statistically significant differences in both behavioral and pathological tests, confidence in these results could be enhanced by increasing n values. Additionally, many r-mTBI studies in the field have focused on the immediate or subacute aftermath (acute [66] to 1–3-month timeframes [35,67,68]), but less is known about extended or chronic recovery. In this study, we deemed it appropriate to test a novel treatment, HOE642, on these shorter, better characterized timeframes before undertaking longer-term studies. Interestingly, r-mTBI mice demonstrate sensorimotor deficits, learning, and working memory impairments at both the 1-year and 24-month timepoints post-r-mTBI. Additionally, r-mTBI animals display higher levels of axonal degeneration (increased APP staining) and persisting neuroinflammation (elevated GFAP and IBA1 staining) in the CC [69,70], with white matter damage and callosal atrophy detected on MRI-DTI one year after r-mTBI [70]. Such prior studies demonstrate that r-mTBI can lead to lifelong degenerative brain damage and suggest that treatment strategies should focus on both acute and chronic timepoints. They also suggest post-r-mTBI-increases in gliosis, axonal degeneration, and motor/cognitive impairment, similar to those we characterize at 60 dpi. Additional studies evaluating the impact of the pharmacological NHE1 protein blockade in the long-term period are warranted.

While antioxidants are often associated with mitigated DNA damage, such as DNA base modifications, single- and double-strand breaks, and the formation of apurinic/apyrimidinic lesions [71], HOE642 has conversely been reported to enhance DNA damage and apoptosis in an acid-tolerating human malignant mesothelioma cell line [72]. Whether post-r-mTBI administration of HOE642 affects DNA damage in injured brains warrants further studies. Finally, FA in DTI is constrained by several limitations. DTI MRI can not distinguish microscopic factors like axon density, caliber, and myelination individually due to the large voxel size in DTI, leading to composite influences on FA values without specific detail on each factor. FA reflects both macroscopic axonal alignment and microscopic axonal properties, yet current techniques struggle to differentiate their distinct impacts, potentially leading to misinterpretations [18]. Our integrated approaches with multiple imaging modalities (histology and MRI DTI) enhanced our understanding of white matter integrity changes after r-mTBI.

## 5. Conclusions

In conclusion, our study demonstrates that r-mTBI induces neurological functional deficits, stimulates neuroinflammation, causes axonal damage, and upregulates the expression of the NHE1 protein (Figure 9). Furthermore, we observed that pharmacological inhibition of the NHE1 protein with HOE642 post-r-mTBI reduces neuroinflammation, oxidative stress, axonal damage, and preserves white matter integrity. Moreover, this treatment attenuates r-mTBI-induced locomotor and cognitive impairments (Figure 9). Thus, our findings identify the NHE1 protein as a potential therapeutic target for r-mTBIs.

## Data Availability

The original contributions presented in this study are included in the article/Supplementary Material; further inquiries can be directed to the corresponding author.

## Supplementary Materials

The following supporting information can be downloaded at: https://www.mdpi.com/article/10.3390/antiox13070836/s1, Figure S1: Negative control staining of sham and r-mTBI mice at 15 dpi. Confocal magnified negative control images from cerebral cortex (CTX), corpus callosum (CC), and hippocampal CA1 regions at 15 dpi; Figure S2: Negative control staining of Veh-r-mTBI and HOE-r-mTBI mice. Confocal magnified negative control images from CTX, CC, and hippocampal CA1 regions at 60 dpi; Figure S3: No apparent differences were detected in locomotor activities, anxiety, and spatial working memory between sham and r-mTBI mice at 40 dpi; Figure S4: No apparent differences were detected in spatial reference memory between sham, Veh-treated and HOE-treated mice at 60 dpi. N = 6 for sham groups, N = 13 for Veh-treated groups, N = 13 for HOE-treated groups. Data are mean ± SEM; Figure S5: Changes of r-mTBI induced axonal damage detected by MRI Diffusion tensor imaging (DTI); Table S1: Number of subjects used per figure; Table S2. List of antibodies used in immunostaining. References [73,74,75,76] are cited in Supplementary Materials.
