# Supplementary material for: NHE1 Protein in Repetitive Mild TBI-Mediated Neuroinflammation and Neurological Function Impairment
Source: Antioxidants (Basel). 2024 Jul 13;13(7):836. doi: 10.3390/antiox13070836 (PMC11274226; doi:10.3390/antiox13070836)
Supplement: Supplementary file 1 [file antioxidants-13-00836-s001.zip › antioxidants-2997664-supplementary.pdf]

Supplementary Information

Materials and Methods

Animals

All animal experiments described in this study were approved by the University of Pittsburgh Institutional Animal Care and Use Committee and adhered to the National Institutes of Health Guide for the Care and Use of Laboratory Animals. Additionally, all studies were reported in accordance with the Animal Research: Reporting In Vivo Experiments (ARRIVE) guidelines [13]. Food and water were provided to animals ad libitum and animals were housed in a temperature-controlled environment with 12/12 hr light–dark cycles. All efforts were made to minimize the number of animals used for experiments and any animal suffering. The number of animals used per figure can be found in **Table S1**.

Adult C57BL/6J wild-type (WT) mice (male, 2–3 months old) were used in this study. For the inhibitor study, the potent NHE1 inhibitor, HOE642 (Cariporide, Sigma-Aldrich, USA), was dissolved at 1 mg/ml in dimethyl sulfoxide (DMSO) stock solution. Immediately before injection, the solution was diluted to 0.025 mg/ml in PBS. For the vehicle control (Veh), 2.5% DMSO in PBS was used. Veh or HOE642 (0.3 mg/kg body weight/day, i.p.) was administered twice daily for 7 days starting at 24 hours after the 5th impact. For transgenic knockout study, *Cx3cr1-CreER*$^{+/-}$ control (Ctrl) mice and *Cx3cr1-CreER*$^{+/-}$*; Nhe1*$^{f/f}$ conditional knockout (*Nhe1* cKO) mice were used as described in our previous study (male and female, 2–3 months old) [9]. At postnatal day 30–40 (P30–P40), both mouse genotypes received tamoxifen (Tam, Sigma-Aldrich, USA) (75 mg/kg body weight/day at a 20 mg/ml concentration in corn oil, intraperitoneally) for 5 consecutive days. To mitigate the impact of *Cx3cr1*$^{+}$ peripheral infiltrating bone marrow-derived

myeloid cells (BMDM) in the CNS, a waiting period of 30 days post-injection was implemented. This period served the dual purpose of clearing Tam and allowing *Cx3cr1*$^{+}$ monocytes to replenish before subjecting the mice to sham or r-mTBI procedures [3-6].

Repetitive mild (r-mTBI) procedures

Adult C57BL/6J WT or transgenic mice were anesthetized using 1.5% isoflurane while core temperature (37 °C) was maintained using a small animal controller pad throughout all procedures. After placing the animal in a stereotaxic frame mounted with a controlled cortical impact (CCI) device (Leica Biosystems, Germany), a small incision was made at the midline of the head to expose the skull. A 5 mm diameter blunt tip was retracted and positioned above the sagittal suture midway between the lambda and bregma. Mice were impacted at 5 m/s, with a strike depth of 1 mm and a dwell time of 200 ms, mimicking an mTBI [14]. Repetitive injuries occurred on days 0, 2, 4, 6, and 8 for a total of 5 impacts with an inter-mTBI interval of 48 hrs. Stepwise changes in righting time and apnea time (indicators of neurological functional recovery) were recorded after each impact to detect animal-to-animal and/or group differences in procedure response [14]. Apnea time refers to the number of seconds before a mouse resumes normal breathing after an impact. After surgery is complete and anesthesia is stopped, mice are placed on their backs under a heating lamp to recover. Righting time refers to the duration of time until the mouse returns to the upright position. Following surgical procedures, the mice were allowed to recover under the heating lamp during a 30 min recovery period. Sham control animals underwent identical procedures without receiving the impact.

Behavioral function tests

Neurological functional impairments in mice were screened in a blinded manner with the rotarod accelerating test, open field test, y-maze spontaneous alternation test, and y-maze novel spatial recognition test. All tests listed were considered reliable for identifying and quantifying sensorimotor and cognitive impairments in rodent models [10,15-17]. Administration of behavioral tests was similar to but slightly modified from previously described procedures [9,10, 16,17].

a) ***Rotarod accelerating test.*** The evaluation of motor coordination and balance changes was conducted using the rotarod apparatus (Model 755; IITC Life Science Inc., USA) as previously described [16,17]. Animals underwent testing both before induction of r-mTBI and at 9, 10, 11, 12, 13, 15, 18, 22, and 29 days post-first-injury (dpi). Initially, animals were familiarized with a stationary rod for 2 min, after which they were placed on a rotating drum that accelerated from 4 to 40 revolutions per min over a 5 min period. The duration the animal remained on the drum was recorded. Three trials were conducted with 5 min breaks between each. The time at which the animal fell off the drum was documented. Pre-operative training was administered once daily for three consecutive days before the first impact.

b) ***Open field test.*** The open field test was employed to identify overall locomotor activity and behavior indicative of anxiety, as previously detailed [16,17]. Animals underwent testing at 30 or 40 dpi. Individual mice were situated in the center of a 50 cm x 50 cm x 50 cm open field chamber and observed for a duration of 30 min using an overhead video tracking system. Parameters such as distance traveled by each animal, its velocity, and the time spent in predefined zones (center, corners, or peripheral areas) were documented.

c) ***Y-maze spontaneous alternation test.*** The Y-maze spontaneous alternation test was employed to evaluate spatial working memory, as outlined in previous studies [16,17]. Animals underwent testing at 32 or 40 dpi. Each mouse was introduced into one arm of the Y-maze and observed for an 8-minute period through an overhead video tracking system. The recorded sequence of arm entries was analyzed using an automated Sequence Analysis Tool macro in Microsoft Excel. For calculating spontaneous alternation, instances were only considered when a mouse consecutively entered three different arms. The spontaneous alternation percentage was determined by subtracting two from the total number of triad spontaneous alternations and expressing it as a percentage of the total arm entries.

d) ***Novel spatial recognition test.*** The spatial reference memory was assessed using the novel spatial recognition test as previously detailed [15,17]. Animals underwent testing at 39 or 40 dpi. The Y-maze, featuring three arms with distinct visual cues of various shapes and colors, served as the testing arena. Each mouse was initially placed in the starting arm and allowed to explore the Y-maze with one arm blocked for a 10 min habituation period. Following this, the animals were returned to their home cage for 5 min and subsequently reintroduced to the maze with all arms open for an additional 5 min. An overhead video tracking system recorded each animal's location, path, and time, while total entries and the time spent (T) in each arm were subjected to analysis. Calculations for the differentiation index (DI) and the recognition index (RI) were $(T_{novel} – T_{familiar}) / T_{total}$ and (T_{novel} / T_{total}), respectively.

MRI and DTI of ex vivo brains

MRI and DTI procedures were similar to those as previously described [9,10]. At 60 dpi, the same cohort of mice that underwent behavioral assessments were humanely euthanized through CO_2

overdose. Subsequently, they underwent transcardial perfusion with ice-cold 0.1M PBS (pH 7.4) followed by an infusion of 4% paraformaldehyde (PFA). The mice were then decapitated, keeping the brains within the skull to prevent anatomical distortion as previously outlined [9,10]. The heads, post-fixed in 4% PFA overnight, were stored in a PBS solution at 4°C. Magnetic Resonance Imaging (MRI) was conducted at 500MHz using a Bruker AV3HD 11.7 T/89 mm vertical bore small animal MRI scanner equipped with a 20 mm quadrature radiofrequency (RF) coil and Paravision 6.10 software (Bruker, Biospin, USA). After positioning and pilot scans, a Diffusion Tensor Imaging (DTI) dataset covering the entire brain was collected using a multislice spin echo sequence with 3 reference and 30 non-collinear diffusion-weighted images. The parameters for DTI included TE/TR = 22/5000 ms, 4 averages, matrix size = 192 x 192 reconstructed to 256 x 256, field of view = 22 x 22mm, 25 axial slices, slice thickness = 1 mm, b-value = 1200 s/mm^2 and Δ/δ = 10/5 ms. Subsequently, the DTI datasets underwent blinded analysis using DSI studio (https://dsi-studio.labsolver.org/). Regions of interest (ROIs) were delineated, segmenting the corpus callosum (CC), hippocampal CA1, internal capsule, and external capsule in both hemispheres from four scanned sections in each brain. Fractional anisotropy (FA), mean diffusivity (MD), axial diffusivity (AD), and radial diffusivity (RD) values were then calculated for each ROI, employing previously described methodology [9,10].

Immunofluorescent staining

Immunofluorescent staining procedures were similar to those used in previous studies [9,10,16,17]. Mice were humanely euthanized and perfused with 4% PFA as described above. After overnight post-fixation in 4% PFA, brains underwent cryoprotection with 30% sucrose.

Coronal sections (25 μm, at the level of 1.46 mm posterior to bregma) were subjected to a 1-hour incubation in a blocking solution (10% normal goat serum and 0.3% Triton X-100 in PBS) at room temperature. Subsequently, they were exposed overnight at 4°C to the following antibodies: mouse monoclonal anti-GFAP (1:200, Cell Signaling Technology, USA), rabbit polyclonal anti-IBA1 (1:200, FUJIFILM Wako Pure Chemical, Japan), mouse monoclonal anti-SMI32 (1:200, Biolegend, USA), rabbit polyclonal anti-APP (1:200, Cell Signaling Technology), rabbit monoclonal anti-NHE1 (1:200, Abcam, USA), mouse monoclonal anti-MAP2 (1:200, Millipore, USA), mouse monoclonal anti-Olig2 (1:200, Millipore, USA), rabbit polyclonal anti-degraded myelin basic protein (1:2000, Millipore, USA), mouse monoclonal anti-NeuN (1:200, Millipore, USA) or rabbit polyclonal anti-phospho-p47phox (Ser304) (1:200, Invitrogen, USA) **(Table S2)**. After three washes in TBS-Triton X-100 (0.3%) for 3 x 10 min, sections were exposed to goat anti-mouse Alexa 488-conjugated IgG (1:200, Invitrogen, USA) and goat anti-rabbit Alexa 546-congugated IgG (1:200, Invitrogen, USA) in blocking solution for 1 hr. Negative controls were established by staining brain sections with secondary antibodies only **(Figs. S1–S2)**. Following three washes, nuclei were stained with DAPI (1:500, Invitrogen, USA) for 15 min at room temperature. Sections were then mounted with Vectashield mounting medium (Vector Laboratories, USA). A minimum of three fluorescent images were captured for each area under a 40x lens using a Nikon A1R inverted confocal laser-scanning microscope (Olympus, Japan). Identical digital imaging acquisition parameters were used, and images were obtained and analyzed in a blinded manner throughout this study. Field intensity was measured within the delineated regions and cell counts were measured by converting into binary images with semi-automated cell counting using consistent threshold parameters for specific cell types.

Results

Table S1. Number of subjects used per figure.

Figure	Number of Subjects
1B	r-mTBI (n = 9)
1C	Sham (n = 9), r-mTBI (n = 9)
1D	Sham (n = 7), r-mTBI (n = 6)
1E	Sham (n = 4), r-mTBI (n = 4)
2B	Sham (n = 4), r-mTBI (n = 5)
2D	Sham (n = 4), r-mTBI (n = 5)
2F	Sham (n = 4), r-mTBI (n = 5)
3C	Sham (n = 4), r-mTBI (n = 5)
4B	Veh-treated (n = 13), HOE-treated (n = 13)
4C	Sham (n = 6), Veh-treated (n = 13), HOE-treated (n = 13)
4D	Sham (n = 6), Veh-treated (n = 13), HOE-treated (n = 13)
4E	Sham (n = 6), Veh-treated (n = 13), HOE-treated (n = 13)
4F	Sham (n = 6), Veh-treated (n = 13), HOE-treated (n = 13)
5C	Sham (n = 4), Veh-treated (n = 6), HOE-treated (n = 6)
6B	Sham (n = 5), Veh-treated (n = 5), HOE-treated (n = 5)
6D	Sham (n = 5), Veh-treated (n = 5), HOE-treated (n = 5)
7B	Sham (n = 6), Veh-treated (n = 5), HOE-treated (n = 5)
7D	Sham (n = 5), Veh-treated (n = 5), HOE-treated (n = 5)
8B	Ctrl-r-mTBI (n = 4), cKO-r-mTBI (n = 5)
8C	Ctrl-r-mTBI (n = 4), cKO-r-mTBI (n = 5)
8E	Ctrl-r-mTBI (n = 4), cKO-r-mTBI (n = 5)
8G	Ctrl-r-mTBI (n = 4), cKO-r-mTBI (n = 5)
S3 A	Sham (n = 5), r-mTBI (n = 3)
S3 B	Sham (n = 5), r-mTBI (n = 3)
S4	Sham (n = 6), Veh-treated (n = 13), HOE-treated (n = 13)
S5	Sham (n = 6), Veh-treated (n = 5), HOE-treated (n = 5)

Table S2. List of antibodies used in immunostaining.

Antibody	Host	Dilution	Company	Catalog No.
GFAP	Mouse	1:200	Cell Signaling Technology	3670S
IBA1	Rabbit	1:200	Wako	019-19741
APP	Rabbit	1:200	Cell Signaling Technology	19389S
SMI32	Mouse	1:200	BioLegend	801701
NHE1	Rabbit	1:200	Abcam	Ab67314
MAP2	Mouse	1:200	Millipore	MAB3418
OLIG2	Mouse	1:200	Millipore	MABN50
NeuN	Mouse	1:200	Millipore	MAB377
Phospho-p47 phox	Rabbit	1:200	Invitrogen	PA5-36773
HNE	Mouse	1:100	Invitrogen	MA5-27570
Degraded Myelin Protein	Rabbit	1:2000	Millipore	AB5864
DAPI	-	1:500	Invitrogen	D1306
Alexa Fluor 488 Goat Anti-Mouse IgG	Goat	1:200	Invitrogen	A11029
Alexa Fluor 546 Goat Anti-Rabbit IgG	Goat	1:200	Invitrogen	A11035

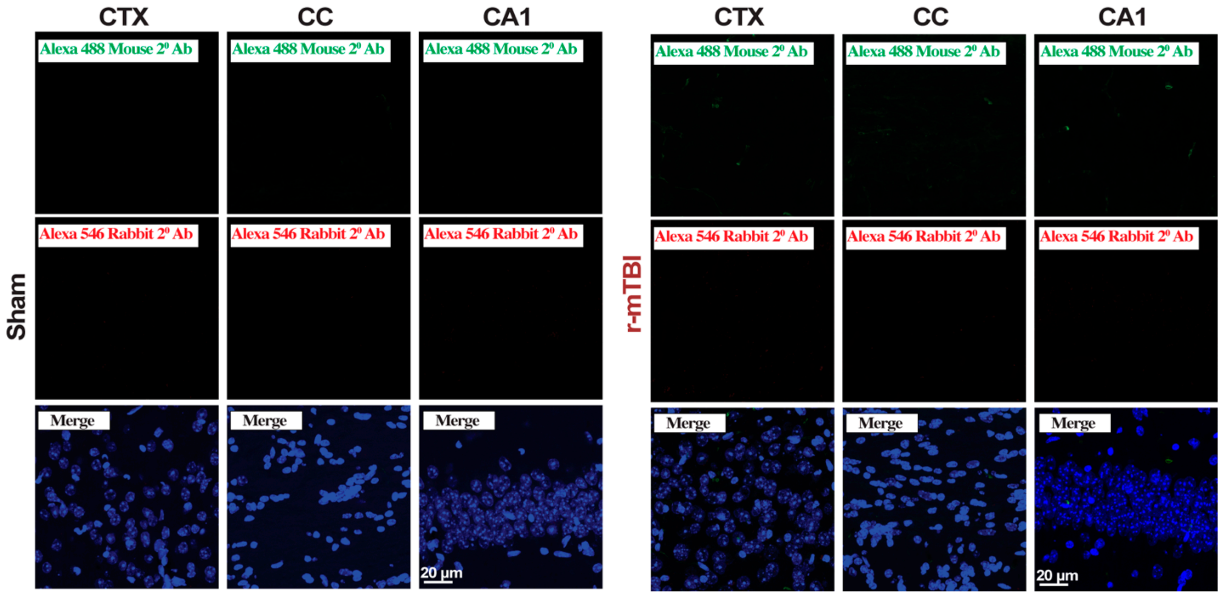

Fig. S1. Negative control staining of sham and r-mTBI mice at 15 dpi. Confocal magnified negative control images from cerebral cortex (CTX), corpus callosum (CC), and hippocampal CA1 regions at 15 dpi.

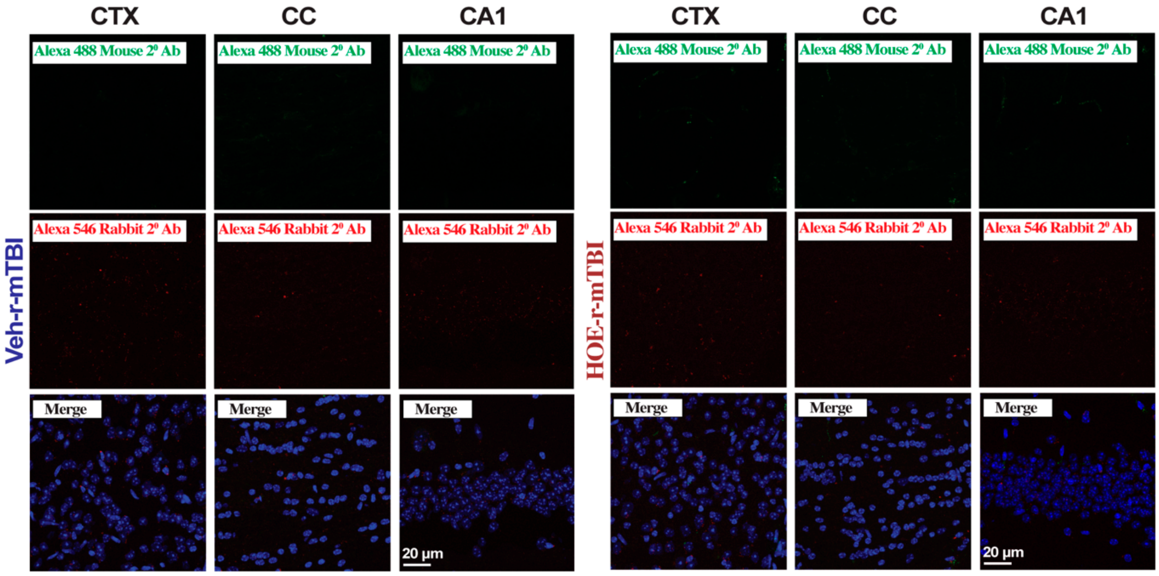

Fig. S2. Negative control staining of Veh-r-mTBI and HOE-r-mTBI mice. Confocal magnified negative control images from CTX, CC, and hippocampal CA1 regions at 60 dpi.

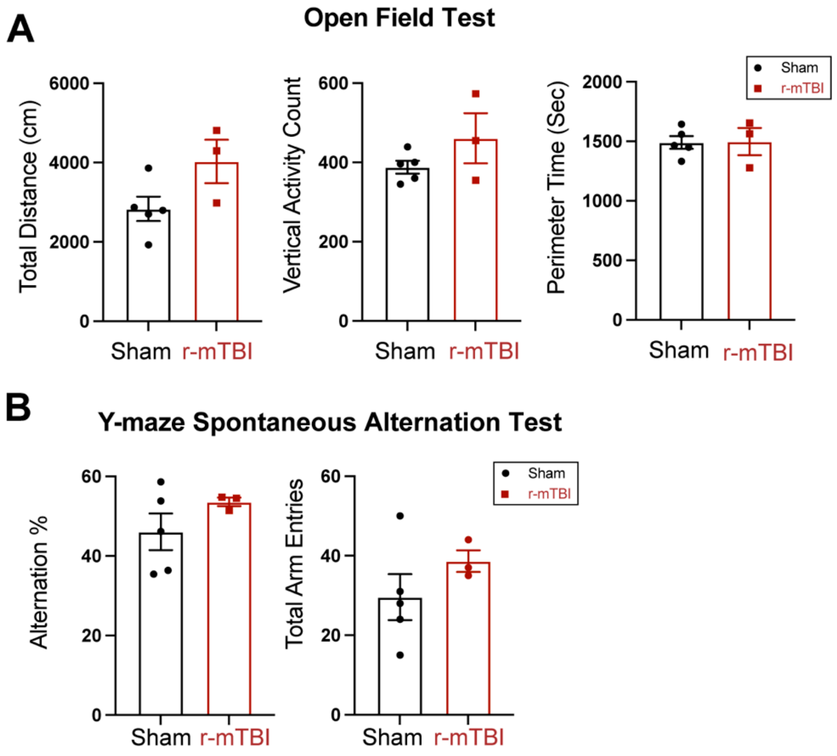

Fig. S3. No apparent differences were detected in locomotor activities, anxiety, and spatial working memory between sham and r-mTBI mice at 40 dpi. **A.** Open field test in the same cohort of mice as in Fig. 1E. **B**. Y-maze spontaneous alternation test in the same cohort of mice as in Fig. 1E. N = 5 for sham groups, N = 3 for r-mTBI groups. Data are mean ± SEM.

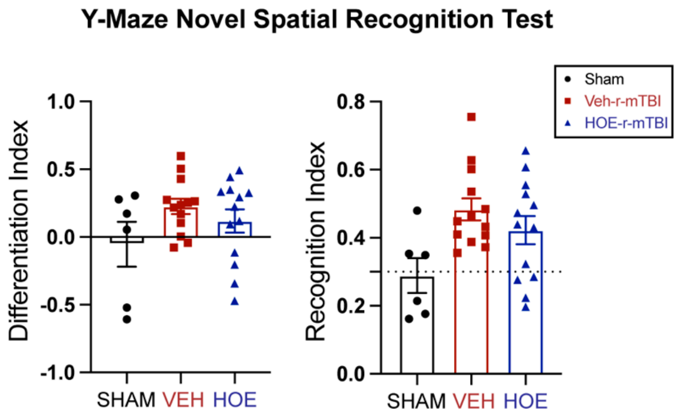

Fig. S4. No apparent differences were detected in spatial reference memory between sham, Veh-treated and HOE-treated mice at 60 dpi. N = 6 for sham groups, N = 13 for Veh-treated groups, N = 13 for HOE-treated groups. Data are mean ± SEM.

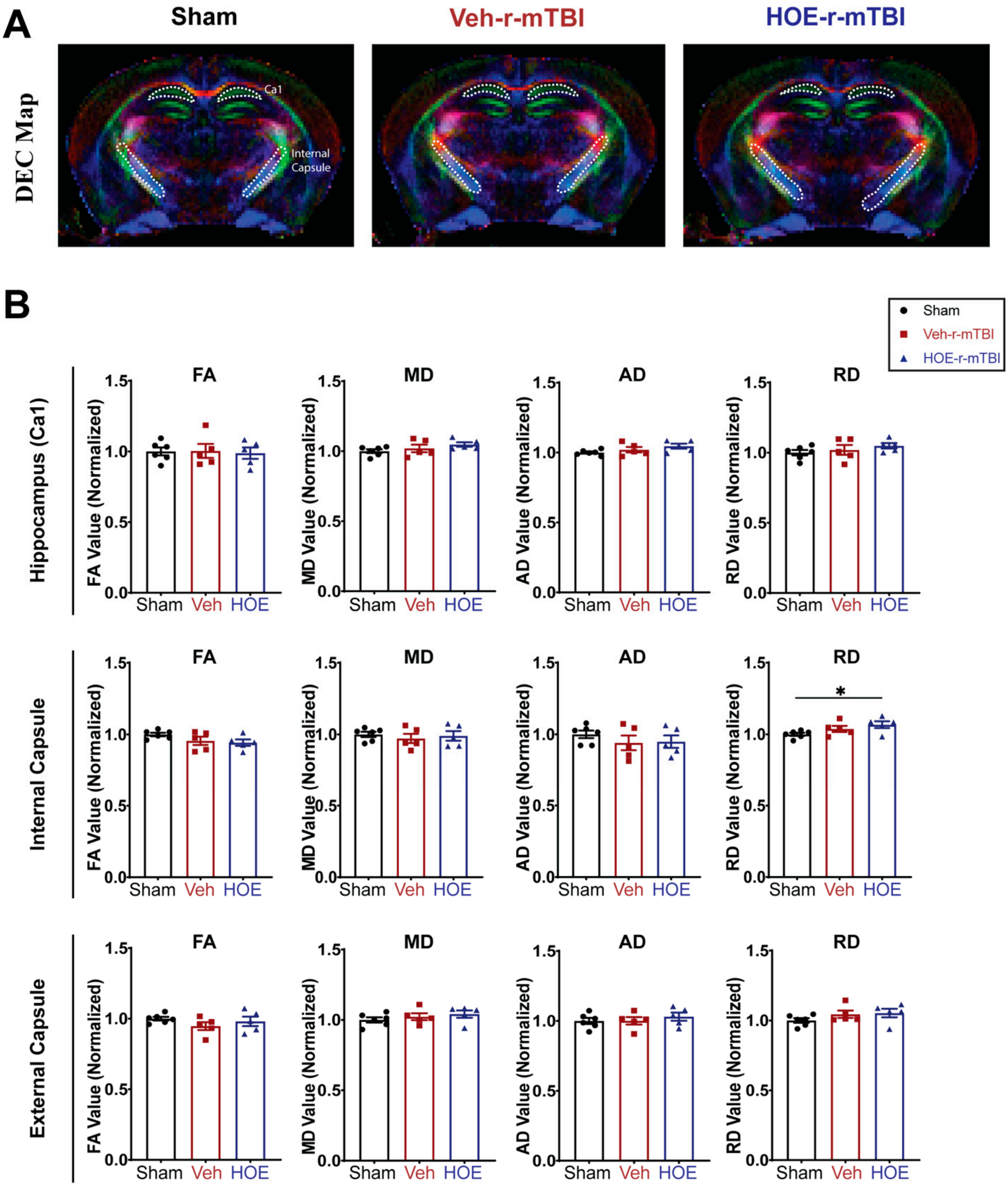

Fig. S5. Changes of r-mTBI-induced axonal damage detected by MRI Diffusion tensor imaging (DTI). **A.** Representative DTI diffusion encoded color (DEC) map of ex vivo brains from sham, Veh-treated and HOE-treated r-mTBI mice at 60 dpi. **B.** Analysis of normalized fractional anisotropy (FA), mean diffusivity (MD), axial diffusivity (AD), and radial diffusivity (RD) values

in the hippocampus (CA1), internal capsule, and external capsule from the same cohort of mice as in A. N = 6 for sham group, N = 5 for Veh-treated group, N = 5 for HOE-treated group. Data are presented as mean ± SEM. * $p < 0.05$.

References

3. Ajami, B.; Bennett, J.L.; Krieger, C.; Tetzlaff, W.; Rossi, F.M. Local self-renewal can sustain CNS microglia maintenance and function throughout adult life. *Nat Neurosci* **2007**, *10*, 1538-1543, doi:10.1038/nn2014.
4. Fogg, D.K.; Sibon, C.; Miled, C.; Jung, S.; Aucouturier, P.; Littman, D.R.; Cumano, A.; Geissmann, F. A clonogenic bone marrow progenitor specific for macrophages and dendritic cells. *Science* **2006**, *311*, 83-87, doi:10.1126/science.1117729.
5. Valny, M.; Honsa, P.; Kirdajova, D.; Kamenik, Z.; Anderova, M. Tamoxifen in the Mouse Brain: Implications for Fate-Mapping Studies Using the Tamoxifen-Inducible Cre-loxP System. *Front Cell Neurosci* **2016**, *10*, 243, doi:10.3389/fncel.2016.00243.
6. Parkhurst, C.N.; Yang, G.; Ninan, I.; Savas, J.N.; Yates, J.R., 3rd; Lafaille, J.J.; Hempstead, B.L.; Littman, D.R.; Gan, W.B. Microglia promote learning-dependent synapse formation through brain-derived neurotrophic factor. *Cell* **2013**, *155*, 1596-1609, doi:10.1016/j.cell.2013.11.030.
